# Report of the Seventh Street Dispensary, Louisville, Ky.

**Published:** 1855-12

**Authors:** David W. Yandell

**Affiliations:** Louisville, Ky.


### ART. III.—REPORT OF THE SEVENTH STREET DISPENSARY, LOUISVILLE, KY.

BY DAVID W. YANDELL, M. D., OF LOUISVILLE, KY.

A SUFFICIENT fund having been contributed by some charitably-disposed citizens of Louisville for the purpose of establishing a Dispensary, an institution of this character was opened on the 13th of September, 1855, under the style of the "Seventh Street Dispensary," and placed under charge of Prof. B. R. Palmer and myself.

Early in October Dr. Palmer found that other duties would oblige him to discontinue his visits to the Dispensary, and shortly afterwards he withdrew temporarily from the institution. Prof. Gross was appointed in his stead, and remained in regular attendance till Dr. Joseph B. Sims, of Trenton, Ky., expressed a willingness to occupy the situation, when the latter gentleman was selected as my assistant in the Dispensary. I feel it my duty to acknowledge in this public way my obligations to Dr. Sims for the valuable aid he has rendered me, both in prescribing for patients at the Dispensary, and in the still more laborious task of visiting many of them at their homes.

The apothecaries' department has been under the control of competent gentlemen selected from my private class.

The annexed tabular statement exhibits the number of persons, with the sex, nativity, disease, and result, who applied at the Dispensary during the two months ending November 13, 1855:

| | |
|---|---|
| Whole number of patients treated | 1,024 |
| Males | 543 |
| Females | 481 |
| | 1,024 |
| Children | 237 |
| Adults | 787 |
| | 1,024 |
| *Results of Treatment.* | |
| Discharged cured | 931 |
| Under treatment | 83 |
| Died | 5 |
| Sent to hospital | 5 |
| | 1,024 |

| *Diseases of those who died.* | |
|---|---|
| Typhoid fever | 2 |
| Congestive chill | 1 |
| Chronic diarrhœa | 1 |
| Convulsions | 1 |
| | 5 |
| *Nativity.* | |
| Ireland | 800 |
| Germany | 141 |
| America | 71 |
| England | 21 |
| Scotland | 1 |
| | 1,024 |

The number affected by each disease, whether attended at the Dispensary or at home, is as follows:

| | | | |
|---|---|---|---|
| Abscess | 1 | Headache | 4 |
| Abscess of jaw | 1 | Injury | 1 |
| Anemia | 3 | Intermittent fever | 851 |
| Amaurosis | 1 | Labor | 5 |
| Asthma | 3 | Marasmus | 1 |
| Amenorrhea | 5 | Myringitis | 1 |
| Bronchitis | 2 | Mammary disease | 1 |
| Blepharitis | 1 | Neuralgia | 5 |
| Boil | 1 | Necrosis of lower jaw | 1 |
| Cystitis | 1 | Onychia maligna | 1 |
| Convulsions | 1 | Opacity of cornea | 1 |
| Consumption | 2 | Prolapsus uteri | 1 |
| Cholera infantum | 2 | Pleurisy | 2 |
| Conjunctivitis | 8 | Profluvia | 1 |
| Colic | 2 | Rheumatism | 2 |
| Catarrh | 4 | Remittent fever | 5 |
| Constipation | 2 | Spanemia | 8 |
| Congestive chill | 1 | Scrofula | 2 |
| Diarrhœa | 25 | Salivation | 1 |
| Dysentery | 12 | Syphilis | 2 |
| Dropsy | 3 | Scarlet fever | 1 |
| Double vision | 1 | Splenalgia | 1 |
| Dysmenorrhea | 1 | Tooth-ache | 4 |
| Dyspepsia | 1 | Tumors | 2 |
| Dislocation of shoulder | 1 | Typhoid fever | 2 |
| Erysipelas | 2 | Tongue-tie | 1 |
| Ear, foreign body in | 1 | Urticaria | 1 |
| Fracture of forearm | 2 | Vertigo | 1 |
| Fracture of arm | 1 | Wound of hand | 1 |
| Gastritis | 1 | Wound of arm | 1 |
| Heart disease | 1 | Whooping cough | 1 |
| Hemorrhoids | 4 | Worms | 1 |
| Hernia | 1 | | |
| Herpes | 1 | | 1,024 |

| | |
|---|---|
| Treated at dispensary | 909 |
| Treated at their homes | 115 |
| | 1,024 |
| The number of prescriptions put up at the Dispensary is | 1,677 |
| The number of prescriptions put up elsewhere | 230 |
| | 1,907 |

It will be seen that the number of cases of intermittent fever has been very large. This, I imagine, would prove equally true of similar tables kept in almost any city in the United States during the same period of time. It is, I believe, the general remark, that this fever has never been so universally prevalent on this continent as during the summer and autumn just ended.

The great majority of the Dispensary patients, who were suffering from intermittent fever, were adults, and many of them had been sick for weeks and often for months before they made application for relief. Nearly all of them had either been prescribed for by physicians of the city, or had used on their own account antiperiodics of every imaginable character. Of the eight hundred and fifty-one cases treated none ended fatally, and at the date of the report eight hundred and nine had been discharged as cured. Of the forty-two who remained under treatment twenty-nine had been registered within seven days—the names of the remaining twelve had either been upon the books but a short time longer, or were excessively intractable cases. The treatment adopted, when the Dispensary was first opened, was with sulphate of quinine. This was soon found to be too costly, and it was decided to make trial of some substitute. The sulphate of quinidine was first used and the results were most satisfactory. The average quantity required to arrest the paroxysm of the disease was from fifteen to twenty grains, in mild tertian cases, and from twenty-five to thirty grains in those of longer standing or greater severity, and in quotidians. The chill was warded off, in many cases, by eight grains of this salt; and where the sufferers were well clad, well housed, and well fed, and not unduly exposed, fifteen grains were almost always sufficient to effect this end.

Creasote was also used in sixty-six cases of intermittent fever, but with very discouraging results. In only twelve of the number was a second chill prevented, and nine of these were children who had suffered but a very short time, and were in every respect comfortably circumstanced. The three remaining cases were adults. In many of those who used the creasote, the remedy was persisted in for ten days without exerting the least influence upon the paroxysm. A small proportion of cases was relieved at the end of five or six days. The usual dose administered to grown persons was from fifteen to thirty drops in the course of the day, given in mucilage.

The number of cases treated by the sulphate of cinchona has not been recorded, though I am sure it cannot be less than between two and three hundred. After reference to the register I incline to think that the latter figures would be nearly correct. And with this substance we have had every reason to be well pleased. It has proven in our hands an exceedingly efficient agent. Indeed, when given in sufficient quantities, it is equal to the relief of almost any ordinary attack of chills. There are instances, of course, in which it fails, just as there are instances in which the mother salt itself fails, but these must be very rare if the medicine be administered in proper amount and the individual treated be at all favorably situated.

According to our experience, both in Dispensary and private practice, relapses occasionally occur under all modes of treatment whatever. So far as an opinion may be based upon the cases treated at the Seventh Street Dispensary by sulphate of cinchona, we do not think that the proportion of relapses has been larger than in the same number treated by either the quinidine or quinine itself. The quantity of sulphate of cinchona used was one-third more than that of the quinidine. The three salts, quinine, quinidine, and sulphate of cinchona, were given both in pill and solution, and were directed to be taken, the first, in doses of about a grain, the second, in doses of about two grains, and the last in doses of about three grains every hour for eight hours before the time of the expected paroxysm. The quantities were not always just as above stated, but varied more or less. These, however, were the regular formulas of the Dispensary. It was very often deemed advisable to unite them with various other substances. In cases that were represented to be very obstinate, that had, for instance, been in the hands of several medical men of the city, without improvement, sulphate of iron and sulphate of copper were added to the prescription—or carbonate of ammonia, or capsicum. And occasionally, where the disease was of long standing, and especially where the interval between the paroxysms was considerable, as seven, or fourteen, or twenty-one days, the extract of bark, in doses of five grains, with one-fouth of a grain of blue vitriol twice or three times a day, was given the preference. These two substances together, and especially when united with a third, the sulphate of iron, yielded highly satisfactory results in the fever when it assumed the seven, fourteen, and twenty-one day type.

In two cases that had proved rebellious to every other means, and in which in the early part of the season had been used, by the advice of a physician of the city, Fowler's solution without benefit, this remedy was exhibited with the effect of arresting the paroxysm at the end of a week. Both individuals were members of the same family, and lived in the outskirts of the city, in a shanty, built upon the bottom of a large pond which had been drained in 1854. Whether their recovery can be attributed to the medicine or to the two facts that cold weather had set in and their systems were almost saturated with iron, is, I think, a question.

The dilute nitric acid was administered in but a single case. This was an example of the twenty-one day form. The cure was complete, the paroxysm never having recurred after the exhibition of the acid.

In the eight hundred and fifty-one cases, whether of recent date or long standing, and of whatever type, the patients were directed, with the smallest number of exceptions, to take conjointly with whatever remedy used, the carbonate of iron and powdered cinchona three times a day, in equal quantities, in doses of from a half to a teaspoonful each. Iron alone or in combination with bark is almost as invariably exhibted in the treatment of intermittent fever at the Seventh Street Dispensary as any of the antiperiodic salts themselves. And we think that much of the success in its management may legitimately be ascribed to this fact.

Save in exceptional cases, it has not been the practice at the Dispensary to premise, as was once the fashion, any opening or preparatory medicine whatever. One of the antiperiodics, and iron, and bark, were at once ordered, and in no instance has anything occurred to cause us to regret this course.

In a large number of instances the individuals who were the subjects of the disease under consideration, were profoundly anemic, and were often suffering from dropsical accumulations, both in the cellular tissue and in some of the cavities of the body. Yet the only change made in the treatment, because of these conditions, was an increase in the quantity of chalybeates. One case, in particular, of general dropsy the result of chills, which now occurs to me, that had been very severely physiced by a city practitioner, who had sent him to the Dispensary in order to get a permit for the hospital, was relieved in less than a fortnight by quinidine as an antiperiodic, and the free use of iron.

The two examples of rheumatism, which appear in the tables, were of the acute articular form, and were treated successfully by

Rochelle salts, after the manner recommended by Dr. Fuller, of London.

The three cases of asthma, two occurring in children, and one in an adult, were greatly relieved during the paroxysms by the gentle inhalation of chloroform.

The case of boil, which was one of boils presenting themselves in various parts of the body, was, in the opinion of the patient, much benefited by the lead plaster applied over the inflamed spots.

The two cases of colic were promptly relieved by the inhalation and internal use of chloroform. The convulsions, in the single case of that affection treated, were controlled by chloroform, though the subject of them died.

The five cases of remittent fever were all treated by the liberal use of cold water in the form of baths, douches, and wet sheets, and the internal administration of ice; and when the febrile reaction and general temperature of the body had been reduced, the rapid introduction into the system of some antiperiodic.

There was one case of simple conjunctivitis, two cases of granular conjunctivitis, and five cases complicated with ulcerations of the cornea—in all eight. The cases of granular lids had been treated in one of the public institutions of the city throughout the greater part of last summer without sensible improvement. They were directed to take full doses of iron, bark, and quinine—the lids were everted and painted daily with a solution of nitrate of silver, ten and fifteen grains to the ounce, and dilute citrine ointment in one case, and the dilute ointment of the red oxide of mercury in the other, applied every night on going to bed. One was relieved in three and the other in six weeks.

Two of the subjects of acute conjunctivitis, with corneal ulcerations, were led to the Dispensary. The inflammation in the eyes of both was excessive. The symptoms were those usual in such cases. The treatment adopted was free scarification of the lids, the bleeding being promoted by warm applications, cups to the temples, mercurial cathartics, and the daily application of a solution of nitrate of silver, ten grains to the ounce, to the palpebral conjunctiva. In order that there might be no question as to the salt being brought into contact with the mucous membrane of the palpebrae, this, after the lids were completely everted, was well dried with lint before the caustic was used. I was the more particular in this latter respect than I would otherwise have been, because it has been much the habit among certain medical men to decry the use of the nitrate of silver in this affection, alleging that where it has apparently been applied with benefit, it was owing to the substance having been prevented from coming in contact with the conjunctiva by this structure being constantly bathed in the mucus and other secretions of the eye. In all the cases in which the caustic solution was employed at the Dispensary, the precaution of drying the mucous membrane was invariably taken, and the solution was used so freely and in such quantities that the most skeptical must have been convinced that the application was most thorough and complete, and could not by any possibility have failed to reach every portion of the conjunctiva, both upon the lids and globe of the eye.

In the two individuals referred to as having been led to the institution, the beneficial effects of the treatment instituted were manifested almost at once. In one every trace of inflammation, both of the conjunctiva and globe of the eye, was removed in six days. The loss of substance consequent upon ulceration was not, of course, repaired so soon. Cicatrization, however, was not long in being effected, and owing mainly to the situation of the ulcers but partly, we must be allowed to think, to the rapidity with which they were healed, the sight of neither eye was in any wise impaired.

Case number two being somewhat less violent in all its main features, was treated with less activity, cups and scarifications being employed but once. To the nitrate of silver we are disposed to give the credit of the cure, which was complete at the expiration of eleven days.

There were three other cases of acute conjunctivitis with ulcerations of the cornea, all occurring in children, two of whom were scrofulous. The only change, however, made in the treatment was as respects the internal medication, which was that adapted to this new constitutional feature. The daily cauterization of the lids with the silver was practiced with as much regularity and I am sure with equal good as in the disease in the adults.

The case of blepharitis was treated after the method recommended by Mr. Critchett, in his lectures in the London Lancet, and after the roots of the eye-lashes were thoroughly cleansed, a single application of the nitrate of silver was sufficient to effect a cure.

The case of double vision is one of some physiological interest. The subject of the affection is an adult Irish laborer, who, having suffered for some weeks with intermittent fever, consulted a quack, who gave him large quantities of a white powder, which, as described by the patient, we think, must have been the sulphate of quinine. After having taken two doses of this substance, he experienced ringing in the ears, and the most intolerable headache, followed the next day by double vision, which continues to this date, December 1st. The expression of the man's eyes is peculiar, and his control over their movements is incomplete. He sees all objects double.

The case of foreign body in the ear is not without interest, and illustrates, in a very striking degree, the value of the speculum in examinations of this organ. The patient, an Irish house-maid, had suffered with ear-ache two years before, for which she was advised to use an application of cotton saturated with laudanum and sweet oil. This she did and with the effect of allaying the pain. But she noticed soon after that she had constant buzzing in the ear, some thickness of hearing, and before long almost total deafness on the affected side. On application at the Dispensary, the patient was unable to hear the ticking of a watch when held directly against the diseased ear. The external auditory canal being thoroughly cleansed, which, however, it required several days to do, the speculum revealed something foreign lying directly upon the tympanum. This was carefully removed, and when examined with a glass, was found to consist of cotton fibres and hardened cerumen, the former of which had doubtless been in the ear from the time of the introduction of the cotton wad two years before. The hearing was promptly and completely restored.

There are two cases reported of consumption. One has passed from under our eyes, and is, we suppose, dead. In the other the disease is in the second stage, and is of a very chronic character. The sufferer, a young English woman, took, while living in Philadelphia, considerable quantities of cod-liver oil. The remedy, however, had grown so repugnant to her that she was unable to continue its use. While under our charge she has used the glycerine instead, and in her own opinion she has been as much benefited by it as she ever was by the oil. It is not offensive, and does not produce nausea or vomiting. The patient has certainly improved slightly in flesh and general appearance since she commenced its use, but whether the change can be fairly ascribed to the remedy or not, I am not prepared to say. No improvement has occurred in the physical signs during this period.

The two cases of typhoid fever, tabled as having died, were not seen till the third week of the disease. The case of congestive chill was death-struck from the start. That of chronic diarrhœa was in the person of a feeble, tuberculous Irish woman, and the case of convulsions, although apparently, at one time, relieved, finally died of cerebral lesion.

Before closing this report I desire to direct the attention of the reader of the Journal to the facts which it contains, as illustrative of the advantages which Louisville offers through this new Charity to the student of medicine. By turning to the tables which it contains it will be observed that the amount and variety of disease presented to the student who visits the Seventh Street Dispensary, are ample for every practical purpose. He cannot fail, during the course of a season, to see examples of every form of disease which he is likely to meet with in practice. It is part of the duty of the physician connected with the institution to assist him in his investigations, to instruct him what to look for, what to observe. When the student is somewhat advanced, cases are entrusted to his care, and then he is required to make a daily report of the symptoms, and of the treatment instituted. The prescriptions being, with few exceptions, put up at the Dispensary by students who are appointed for the purpose, and who serve, for a given length of time, the embryo physician has abundant opportunity to study both the articles of his future armamentarium, the manner of compounding them, and their therapeutic application.

During the winter season, on six days, and during the remainder of the year, on three days in the week, clincical lessons, of a strictly practical character, are given at the Dispensary.

The institution may be considered a permanent one, and during the approaching session of the Legislature a charter will be obtained for it, in order to enlarge the basis of its operations.

*December* 1, 1855.

